# Monolithically, widely tunable quantum cascade lasers based on a heterogeneous active region design

**DOI:** 10.1038/srep25213

**Published:** 2016-06-08

**Authors:** Wenjia Zhou, Neelanjan Bandyopadhyay, Donghai Wu, Ryan McClintock, Manijeh Razeghi

**Affiliations:** 1 Center for Quantum Devices, Department of Electrical Engineering and Computer Science, Northwestern University, Evanston, IL 60208, USA

## Abstract

Quantum cascade lasers (QCLs) have become important laser sources for accessing the mid-infrared (mid-IR) spectral range, achieving watt-level continuous wave operation in a compact package at room temperature. However, up to now, wavelength tuning, which is desirable for most applications, has relied on external cavity feedback or exhibited a limited monolithic tuning range. Here we demonstrate a widely tunable QCL source over the 6.2 to 9.1 μm wavelength range with a single emitting aperture by integrating an eight-laser sampled grating distributed feedback laser array with an on-chip beam combiner. The laser gain medium is based on a five-core heterogeneous QCL wafer. A compact tunable laser system was built to drive the individual lasers within the array and produce any desired wavelength within the available spectral range. A rapid, broadband spectral measurement (520 cm^−1^) of methane using the tunable laser source shows excellent agreement to a measurement made using a standard low-speed infrared spectrometer. This monolithic, widely tunable laser technology is compact, with no moving parts, and will open new opportunities for MIR spectroscopy and chemical sensing.

Many chemicals, like N_2_O, CH_4_, CO_2_, have their signature absorption spectra in the mid-infrared (mid-IR) wavelength range^1^. For chemical-sensing based applications, including medical diagnostics, explosive detection and industrial process monitoring, a portable laser emitting in a wide wavelength range is highly desired. Quantum cascade lasers^2^ (QCLs) have been the leading semiconductor laser sources in the mid-IR wavelength range, thanks to the rapid developments in power, wall-plug efficiency, and single mode operation in the last few years^3^,^4^,^5^,^6^. The recently demonstrated broadband QCL, with a gain bandwidth of ~5.9–10.9 μm, has incredible potential for mid-IR spectroscopy^7^.

Broadband sensing applications require not only a broadband gain medium but also a robust tuning mechanism that allows selecting any wavelength at will in a wide wavelength range. However, the frequency tuning exhibited by QCL devices has either relied on external cavity feedback or has exhibited a limited monolithic tuning range^8^,^9^,^10^,^11^,^12^,^13^. As has been demonstrated at telecommunications wavelengths near λ = 1.55 μm^14^,^15^, multi-channel wavelength-selectable distributed feedback (DFB) laser arrays can be monolithically integrated with an optical beam combiner to realize wide tuning on a single chip. The benefit of the beam combiner is that all wavelengths come out of a single output aperture, along with a compact size and fast electric tuning. In the case of QCLs, the tuning bandwidth is much wider than that of the near infrared lasers. Fortunately, the DFB lasers can be replaced by sampled grating DFB (SGDFB) lasers, which have been demonstrated to extend the tunability of a QCL by one order of magnitude^10^. Here we integrate a broadband QCL wafer with a SGDFB laser array and an on-chip beam combiner. A monolithic tuning of 6.2–9.1 μm is achieved. A compact tunable laser system is built to drive the individual lasers and implement wavelength scanning. Using this system, we demonstrate rapid broadband absorption spectroscopy with no moving parts.

## Results

### Broadband heterogeneous quantum cascade laser core

**Figure 1:**
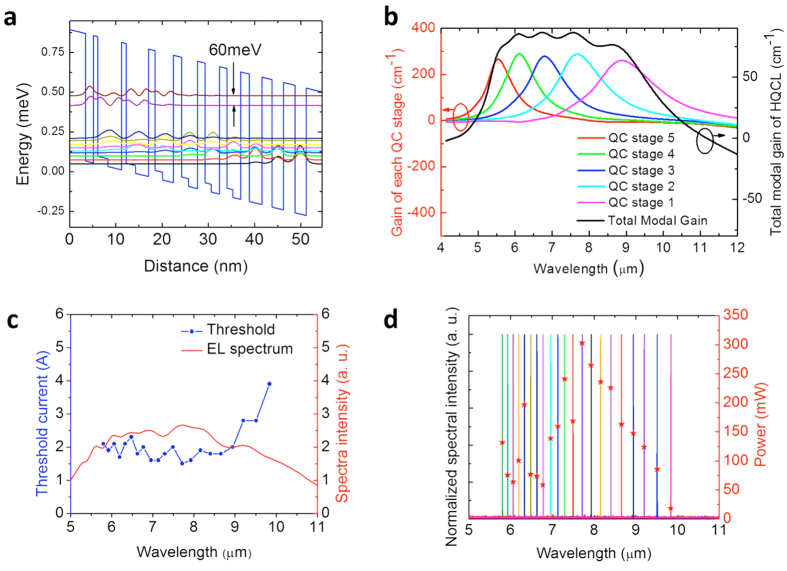
Heterogeneous quantum cascade laser core design. (**a**) Conduction band diagram and relevant wavefunctions for one emitting stage of a quantum cascade laser based on the Al_0.63_In_0.37_As/Ga_0.35_In_0.65_As/Ga_0.47_In_0.53_As material system. (**b**) Simulated gain curve of a five-core heterogenous QCL. (**c**) Overlay plots of EL curve and single mode DFB laser array threshold as a function of wavelength for the five-core QCL design. (**d**) Overlay plot of compiled emission spectra and single mode peak power as a function of wavelength.

The laser core design is based on the same methodology described in ref. 7. Five QCL sub-cores with peak gain energies spaced ~20 meV apart are used to form the heterogeneous QCL core that targets the 6–10 μm wavelength range. The designed laser cores are based on the strain balanced Al_0.63_In_0.37_As/Ga_0.35_In_0.65_As/Ga_0.47_In_0.53_As material system as shown in Fig. 1a. The maximum band offset of this material system is estimated to be ~800 meV and was shown to have good performance across the 6–10 μm wavelength range^16^. By varying only the relative layer thicknesses all emitters can be incorporated into a heterogeneous QCL core in a single growth run. When operated at the resonant field, current densities of all QC stages are nominally ~10 kA/cm^2^, which is accomplished by adjusting the doping concentration and upper laser level electron lifetime. As shown in Fig. 1b, the gain curve of the heterogeneous QCL is predicted to be broad and flat, which allows selection of the laser emitting wavelength over a wide range with an appropriate feedback mechanism. In order to characterize the gain bandwidth and flatness of the designed laser, the grown wafer is characterized with a DFB laser array in the same way as in ref. 7. Electroluminescence (EL) of the heterogeneous QCL wafer is shown in Fig. 1c. The EL spectrum has a full width at half maximum (FWHM) of 1050 cm^−1^ with a center near 1420 cm^−1^. As shown in Fig. 1d, single mode DFB emission between 5.8 to 9.9 μm is obtained from a twenty-one DFB laser array of 3 mm long with both facets AR-coated with 1300 nm Y_2_O_3_. The residual reflectivity is below 7% across the 6–10 μm range with a minimum of 1.1% at 8 μm. The current threshold is relatively flat between 5.8 and 9.0 μm and increases rapidly beyond 9.0 μm. The maximum power output is about 300 mW per facet at a wavelength of 7.7 μm, and most DFB lasers have power output above 100 mW.

### Monolithically tunable QCL source with a single output

**Figure 2:**
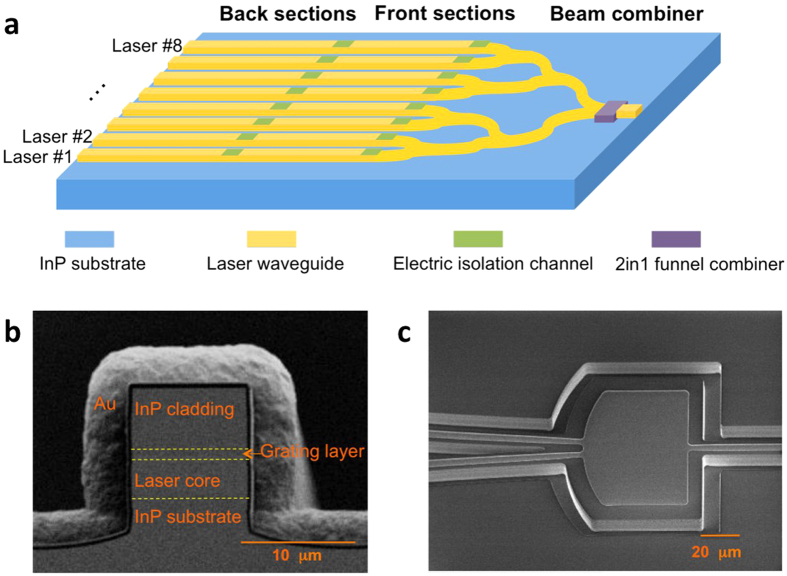
Monolithically tunable QCL device design. (**a**) Schematic of the wavelength tunable QCL source with monolithically integrated SGDFB laser array and beam combiner. Scanning electron microscope (SEM) images of (**b**) cleaved laser facet and (**c**), two-in-one funnel combiner with Si_3_N_4_ passivation and openings on top of the waveguide.

The main limitation of the demonstrated DFB laser array is that it has multiple output apertures and limited tuning for each discrete single mode. To fill in the spectral gaps and realize a single output aperture, the twenty-one DFB QCLs are converted to an eight SGDFB QCL array and a beam combiner (see Methods section). Figure 2a shows the schematic of the integrated device. Two electric isolation channels are defined. One isolates the two SGDFB sections, and the other isolates the beam combiner section from the SGDFB laser array. Each SGDFB laser is 5.5 mm long with a ridge width of 10 μm. The general SGDFB laser source is comprised of a series of short gratings (with grating period and number of periods defined as *Λ*_*g*_ and *N*_*g*_, respectively) periodically sampled on the two sections with two different sampling periods *Z*_1_ and *Z*_2_. For this demonstration, both sections of every SGDFB laser are sampled with *N*_*g*_ = 20 for 16 times, and *Λ*_*g*_ranges from 0.97 to 1.40 μm from laser #1 to #8. As only a short region is patterned, the 750 nm grating layer is completely etched to provide a maximum coupling strength. The sampling periods *Z*_1_ = 161.8 μm and *Z*_2_ = 171.5 μm are used for the front and back sections, respectively. The ridge waveguide is dry-etched by an inductively coupled plasma (ICP) and the cross section is shown in Fig. 2b. The plasma etching produces vertical sidewalls and precise shape for curved waveguides. The separation between each laser is as narrow as 100 μm, which is chosen to enable direct wire bonding. The eight lasers are routed to a single output using a three-stage tree array Y-junction and S-bend waveguides. Given the combiner section length of 3 mm, the bending radius of the S-bend waveguide is 1800 μm. A critical consideration for the beam combiner design is the preservation of the fundamental transverse mode. Higher order mode operation has been reported for Y-junction QCLs^17^. As a solution, we replaced the last Y-junction with a two-in-one funnel combiner^15^. As shown in Fig. 2c, the funnel combiner is designed to partially filter out high order modes that may be present at the input waveguide. Mode filtering improves as the relative angle between the input and output waveguide decreases, though there is some tradeoff in power transmission. For this demonstration, the insertion angle is set at 3.5° with a funnel combiner length and width of 82 μm and 100 μm, respectively.

**Figure 3:**
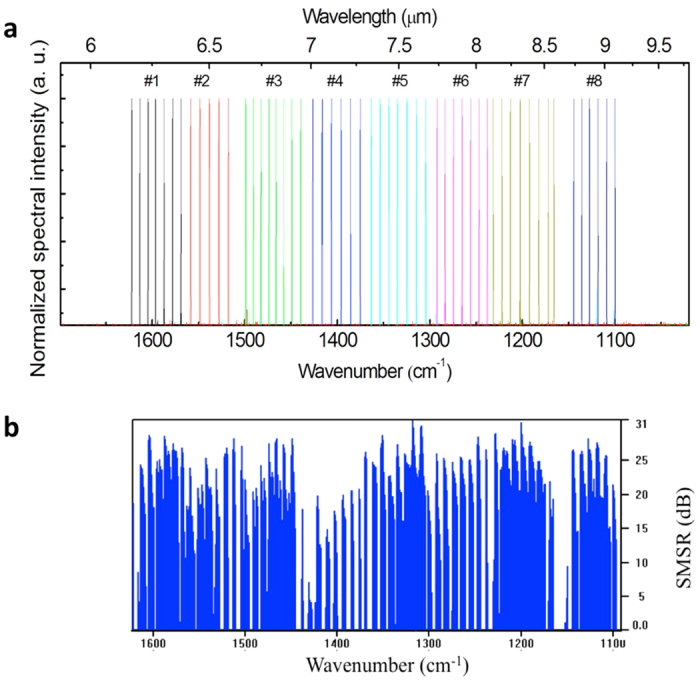
Spectral tuning characterization of the monolithic laser source. (**a**) Compiled Vernier tuning spectra of the eight SGDFB laser array emitting from a single aperture using manual measurement. The tuning ranges from 1100 to 1622 cm^−1^. There are eight sets of peaks, represented in different colors, corresponding to laser #1 through #8, respectively. (**b**) Side mode suppression ratio (SMSR) as a function of wavenumber of the single mode emission conditions selected from automatic continuous tuning measurements.

In order to use the beam combiner as a single pass amplifier, after cleaving, the front facet is AR-coated with 1300 nm Y_2_O_3_. Tuning spectrum measurements were carried out on a thermo-electrically cooled (TEC) stage at 15 °C using a Fourier transform infrared spectrometer (FTIR) at a resolution of 0.125 cm^−1^ (see Methods section). One laser at a time in the SGDFB laser array was selectively biased, and the spectrum of the beam coming out of the single aperture beam combiner was measured. Synchronized pulse currents with 100 ns pulse width and 5% duty cycle (500 kHz repetition rate) were first applied, with separate drivers, to both sections of the selected SGDFB laser to reach threshold (~21 V). The voltage was kept the same on both sections and was further increased until single mode emission could be maintained (~24 V, I = 1.35I_th_). Because the beam combiner section has the same gain material as the laser sections, a third synchronized pulsed current was applied to the beam combiner section for power amplification. For the current demonstration, single mode emission can be maintained, with high side mode suppression ratios (SMSR), for a combiner voltage of ~19 V. Wavelength tuning is realized by applying additional independent continuous wave (CW) currents to the two SGDFB laser sections. The injected CW currents locally change the section temperature, which leads to a change of the effective refractive index. The tuning of SGDFB lasers is the combination of steplike Vernier tuning, which depends on the CW current difference between the two sections, and the continuous tuning of each emission wavelength, achieved by changing the current in the two sections proportionally. Based on the SGDFB grating design, the estimated Vernier tuning step size is 9.7 cm^−1^ and 9.2 cm^−1^ with front and back section tuning, respectively. Figure 3a shows the combined Vernier tuning of 520 cm^−1^ between 6.2 and 9.1 μm of the beam coming out of the single aperture beam combiner. The tuning of each laser is 64, 52, 60, 52, 58, 55, 65 and 45 cm^−1^ for lasers #1 through #8, respectively. The power of each single mode emission line is measured with a thermopile placed in front of the beam combiner facet. With the beam combiner amplification, an optical output power of several milliwatts has been achieved. The highest peak power of 5 mW is from laser #6, where the emission wavelength is close to a maximum in the gain curve. The relatively low power of the SGDFB laser compared to the DFB laser array is due to a significantly higher threshold (I_th,SGDFB_ = 2.2I_th,DFB_) and the coupling losses in the beam combiner region. Potentially, the power performance can be improved with better thermal management or stronger grating coupling, which will decrease the threshold current. Also, the output power is limited by the low beam combiner current due to multi-mode lasing at high current conditions. Reducing facet reflectivity, using multi-layer AR-coating or angled facet amplifier^18^, can increase the multi-mode lasing threshold. With future development, there is substantial room for power improvement by reducing the facet reflectivity. Even more, the power output across the entire tuning range can be uniformed by adjusting the beam combiner current.

As mentioned above, the Vernier tuning mechanism is not continuous, with a step of 9.2–9.7 cm^−1^ corresponding to the frequency spacing of the supermodes formed by the sampled gratings. Continuous tuning, however, can help fill in these gaps. Figure 3b shows the side mode suppression ratio (SMSR) of all the compiled single mode spectra measured from the beam combiner facet selected from automatic continuous tuning measurement by changing the front and back section CW currents. Most spectra have a side mode suppression ratio (SMSR) above 20dB, which suggests these wavelengths are suitable for spectroscopy applications. However, full wavelength coverage between 6.2 and 9.1 μm is not achieved with these lasers, because the continuous tuning of a single mode is, at this time, limited to a maximum of 5.8 cm^−1^. Based on the temperature-dependent spectral measurement of a DFB laser, which is processed from the same wafer, by increasing the laser stage temperature, a tuning coefficient of 0.1 cm^−1^/K is estimated. This means that a ~60-degree increase in temperature makes the laser unable to reach threshold. With future development, the continuous tuning gaps can be bridged by decreasing the supermode space by increasing the sampling period length Z_1_ and Z_2_.

### Broadband absorption spectroscopy of methane

**Figure 4:**
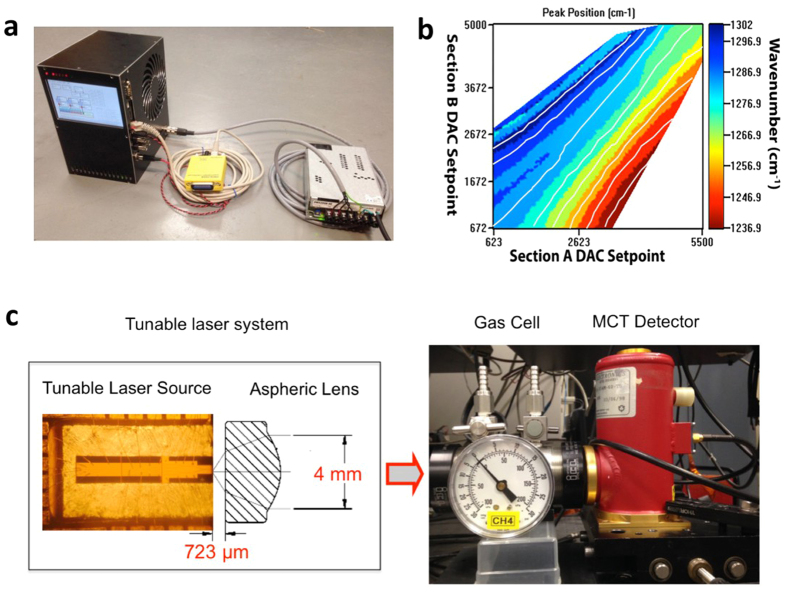
Tunable laser system for chemical sensing. (**a**) Picture of the tunable laser system with GPIB transceiver and power supply. The main system measures 127 mm × 203 mm × 184 mm. (**b**) Colormap of Laser #6 in the array, showing the peak position as a function of front and back section digital to analog (DAC) settings. The heavy grey lines show the calibration paths for this laser. (**c**) Optical microscope picture of the tunable laser array with a schematic view of the aspheric lens used to collimate the output beam (left). The laser chip is 2 mm wide and 8.5 mm long, and the output beam diameter is ~4 mm. Also shown are the 15 cm long gas cell filled with methane and the liquid nitrogen cooled mercury cadmium telluride (MCT) detector used for measurement (right).

**Figure 5:**
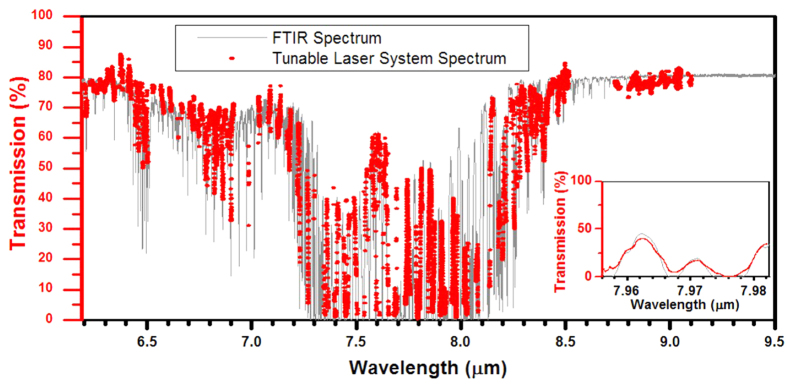
Broadband absorption spectroscopy of methane. Comparison of the spectrum measured with the tunable laser system (red dots) to the expected spectrum measured with the FTIR. The inset shows a zoomed in region showing the excellent agreement.

In order to automatically scan across all available wavelengths for chemical spectroscopy, a self-contained tunable laser system was designed and built as shown in Fig. 4a. This system works off of a single 48 V DC power supply and contains all of the electronics necessary to drive the individual lasers within the array and coordinate the driving of the laser array to produce a desired wavelength (see Methods section). The laser beam comes out as a single collimated output from the front of the system. The system was calibrated using custom automatic calibration software, which records the laser output spectrum with a FTIR as a function of tuning current combinations. For each of the lasers in the array, a colormap is made showing the peak wavelength as function of front and back section current set points. This is then used to generate optimized paths through the data and create a table of pre-calibrated scan paths, as shown in Fig. 4b. The software is then able to interpolate along these paths with a resolution of better than 0.1 nm. In order to use the system for wavelength scanning, a wavelength region of interest is selected and a scan is generated by specifying start, stop, and step conditions. In order to support high speed scanning, each discrete scan-state is downloaded to a dedicated random access memory (RAM) chip on the control board. A crystal oscillator and a phase-locked loop and frequency divider within the control field programmable gate array (FPGA) are then used to step through the states stored in the RAM. The electronic hardware supports a scan rate of up to 32 kHz. Figure 4c shows the setup for the absorption spectroscopy measurement, which consists of the tunable laser system, the gas cell, and a cryogenic mercury cadmium telluride (MCT) detector (see Methods section). The gas cell is filled with methane at 1 atmosphere. In order to measure the broadband absorption spectroscopy of methane, the tunable laser system was scanned from 6.2 μm to 9.1 μm with a step size of 0.1 nm at a scan rate of 512 Hz, which is limited by the maximum data-sampling rate of our lock-in amplifier. Because the tunable device has a single output and a lens installed, we can scan over all eight lasers in the array without needing realignment. The gas cell was first scanned with a vacuum FTIR in order to obtain a reference spectrum. Figure 5 shows a comparison of the reference spectrum (grey) to that measured with the tunable laser system (red points). In general, the tunable laser system is able to accurately measure the spectrum of methane. In particular, the inset of Fig. 5 shows excellent agreement with some of the fine spectral features.

## Discussion

In conclusion, we demonstrated a tunable, broadband QCL source that emits at wavelengths from 6.2 μm to 9.1 μm (520 cm^−1^) using an eight-laser SGDFB array integrated with an on-chip beam combiner. A compact tunable laser system was built to drive the individual lasers within the array with the conditions necessary to produce any accessible wavelength. The system is able to do fast wavelength scanning without being limited by the driving hardware electronics. The spectral measurement of methane from 6.2 μm to 9.1 μm shows excellent agreement with FTIR measurement. This broadly tunable QCL sources with no moving parts will open new opportunities for mid-IR spectroscopy and chemical sensing.

## Methods

### Growth and fabrication

The QCL structure presented in this work is based on the Al_0.63_In_0.37_As/Ga_0.35_In_0.65_As/Ga_0.47_In_0.53_As material system grown by gas-source molecular beam epitaxy on a semi-insulating InP substrate. The layer sequence and the waveguide doping are as follows: 3-μm InP buffer layer (Si, ~2 × 10^16^ cm^−3^), five-core heterogeneous laser core, 100-nm InP spacer layer (Si, ~2 × 10^16^ cm^−3^), and 750-nm GaInAs grating layer (Si, ~2  × 10^16^ cm^−3^). The grating is defined with e-beam lithography and dry etching on the GaInAs grating layer. After the grating patterning, a regrowth of 4-μm low-doped cladding (Si, ~2 × 10^16^ cm^−3^) and 1-μm high-doped cap layer (Si, ~5 × 10^18^ cm^−3^) is performed by low pressure metalorganic chemical vapor deposition (MOCVD). Two isolation channels were etched between the adjacent laser sections. These channels were etched 2.0 μm deep through an 80 μm wide mask into the InP cap and cladding layers. Subsequently, the epilayers were processed into double channel waveguides with a ridge width of 10 μm by plasma etching. Before the formation of top metal contact, a 500 nm thick Si_3_N_4_ layer was deposited and etched through on top of the laser waveguide to define current injection. The top contact is then formed by the evaporation of Ti/Au, followed by a lift-off process and the electroplating of a thick gold layer of 3 μm. After polishing the substrate to 150 μm, a AuGe/Ni/Au bottom contact is evaporated. The sample was cleaved into 8.5 mm long bars and AR coated with 1.3-μm Y_2_O_3_ on the beam combiner facet. The device was mounted epilayer-side up on a copper heat sink with indium solder for testing.

### Device Testing

All measurements were performed at room temperature. Spectral measurements were performed with a Bruker Fourier transform infrared (FTIR) spectrometer at a resolution of 0.125 cm^−1^. The lasers operate in pulsed mode (100 ns pulse width, 500 kHz repetition rate), with the addition of DC biases to different sections to tune the laser frequencies. Three independent drivers were used to inject the pulse/DC currents for spectral and power measurements. Output mid-IR power in pulsed mode was measured using a calibrated thermopile detector for the average power and the peak power was calculated from the measured average power and the known duty cycle.

### Tunable laser system

Within the system, the laser array is mounted on a thermo-electrically cooled (TEC) stage held at a stable heatsink temperature of 15 °C. This stage is mounted on invar-alloy posts for maximum laser pointing stability. The lasers within the array share a common cathode which is grounded to the heatsink. The system has three separate positive polarity laser drivers, each with a variable DC current and pulsed resistively loaded AC voltage. High-efficiency buck-converter power supplies (Texas Instruments TPS54360) with either current or voltage feedback being mixed with a digital to analog converter (DAC) signal are used to dynamically control the DC current and pulsed voltage of each driver. Two 1 × 8 multiplexers consisting of p-FETs (Vishay Si7309DN) select the laser array pair to be driven. The pulsed voltage is applied to the selected laser pair and the beam combiner via n-FETs (Vishay SiS892ADN). All of the FETs are driven by an embedded field programmable gate array (FPGA) via isolated gate drivers (Analog Devices ADuM4223). A separate FPGA controls the DAC settings and monitors the power supplies. The system uses an ARM cortex A9 based embedded single board computer (Toradex Colibri T30) as the main controller.

### Spectroscopy measurement setup

The photoconductive detector is biased with 30 mA of DC current though a bias tee, and the output is connected to a high-speed lock-in amplifier (Stanford Research Systems SR488). A 500 kHz synchronization pulse locked to the laser pulsing is used as the reference input to the lock-in amplifier. An external measurement trigger output from the laser system is used to trigger the lock-in amplifier to record a data point into the lock-in’s internal storage. The scanning and download of data after a scan are controlled remotely via a custom LabVIEW program. The tunable laser system can scan between a wavelength range with a designated step size, first with an open beam and then with the methane filled gas cell in the beam path. After correcting for the reflectivity of the windows, these two scans were then divided by each other to reveal the transmission spectrum of the methane.

## Additional Information

**How to cite this article**: Zhou, W. *et al*. Monolithically, widely tunable quantum cascade lasers based on a heterogeneous active region design. *Sci. Rep*. **6**, 25213; doi: 10.1038/srep25213 (2016).

## References

[b1] Li J. S., Chen W. & Fischer H., Quantum cascade laser spectrometry techniques: a new trend in atmospheric chemistry. Appl. Spectrosc. Rev. 48, 523–559 (2013).

[b2] Faist J. . Quantum cascade laser. Science 264, 553–556 (1994). 17732739 10.1126/science.264.5158.553

[b3] Razeghi M. . Quantum cascade lasers: from tool to product. Opt. Express 23, 8462–8475 (2015). 25968685 10.1364/OE.23.008462

[b4] Bai Y., Slivken S., Darvish S. R. & Razeghi M. Room temperature continuous wave operation of quantum cascade lasers with 12.5% wall plug efficiency. Appl. Phys. Lett. 93, 021103 (2008).

[b5] Bai Y., Bandyopadhyay N., Tsao S., Slivken S. & Razeghi M. Room temperature quantum cascade lasers with 27% wall plug efficiency. Appl. Phys. Lett. 98, 181102 (2011).

[b6] Lu Q. Y., Bai Y., Bandyopadhyay N., Slivken S. & Razeghi M., Room-temperature continuous wave operation of distributed feedback quantum cascade lasers with watt-level power output. Appl. Phys. Lett. 97, 231119 (2010).

[b7] Bandyopadhyay N., Chen M., Sengupta S., Slivken S. & Razeghi M. Ultra-broadband quantum cascade laser, tunable over 760 cm^−1^, with balanced gain. Opt. Express 23, 21159–21164 (2015). 26367965 10.1364/OE.23.021159

[b8] Hugi A. . External cavity quantum cascade laser tunable from 7.6 to 11.4 μm. Appl. Phys. Lett. 95, 061103 (2009).

[b9] Lyakh A., Barron-Jimenez R., Dunayevskiy I., Go R. & Patel C. K. N. External cavity quantum cascade lasers with ultra rapid acousto-optic tuning. Appl. Phys. Lett. 106, 141101 (2015).

[b10] Slivken S. . Sampled grating, distributed feedback quantum cascade lasers with broad tunability and continuous operation at room temperature. Appl. Phys. Lett. 100, 261112 (2012).

[b11] Slivken S. . Dual section quantum cascade lasers with wide electrical tuning. Proc. SPIE 8631, 86310P (2013).

[b12] Kalchmair S. . High tuning stability of sampled grating quantum cascade lasers. Opt. Express 23, 15734–15747 (2015). 26193552 10.1364/OE.23.015734

[b13] Hu X. N. . Broadly continuously tunable slot waveguide quantum cascade lasers based on a continuum-to-continuum active region design. Appl. Phys. Lett. 107, 111110 (2015).

[b14] Kudo K. . 1.55-μm wavelength-selectable microarray DFB-LD’s with monolithically integrated MMI combiner, SOA, and EA-Modulator. IEEE Photon. Technol. Lett. 12, 242–244 (2000).

[b15] Ishii H. . Widely wavelength-tunable DFB laser array integrated with funnel combiner. IEEE J. Sel. Top. Quantum Electron. 13, 1089–1094 (2007).

[b16] Bandyopadhyay N., Bai Y., Slivken S. & Razeghi M., High power operation of λ∼ 5.2–11 μm strain balanced quantum cascade lasers based on the same material composition. Appl. Phys. Lett. 105, 071106 (2014).

[b17] Lyakh A., Maulini R., Tsekoun A., Go R. & Patel C. K. N. Continuous wave operation of buried heterostructure 4.6 μm quantum cascade laser Y-junctions and tree arrays. Opt. Express, 22, 1203–1208 (2014). 24515081 10.1364/OE.22.001203

[b18] Beck M., Jonathon B., Greg F., Larry C. & Steven P. D. Design of sampled grating DBR lasers with integrated semiconductor optical amplifiers. IEEE Photon. Technol. Lett. 12, 762–764 (2000).

